# No Relationship between Embryo Morphology and Successful Derivation of Human Embryonic Stem Cell Lines

**DOI:** 10.1371/journal.pone.0015329

**Published:** 2010-12-31

**Authors:** Susanne Ström, Kenny Rodriguez-Wallberg, Frida Holm, Rosita Bergström, Linda Eklund, Anne-Marie Strömberg, Outi Hovatta

**Affiliations:** 1 Department of Clinical Science, Intervention and Technology, Karolinska Institutet, Stockholm, Sweden; 2 Fertility Unit, Karolinska University Hospital, Huddinge, Sweden; Massachusetts General Hospital, United States of America

## Abstract

**Background:**

The large number (30) of permanent human embryonic stem cell (hESC) lines and additional 29 which did not continue growing, in our laboratory at Karolinska Institutet have given us a possibility to analyse the relationship between embryo morphology and the success of derivation of hESC lines. The derivation method has been improved during the period 2002–2009, towards fewer xeno-components. Embryo quality is important as regards the likelihood of pregnancy, but there is little information regarding likelihood of stem cell derivation.

**Methods:**

We evaluated the relationship of pronuclear zygote stage, the score based on embryo morphology and developmental rate at cleavage state, and the morphology of the blastocyst at the time of donation to stem cell research, to see how they correlated to successful establishment of new hESC lines.

**Results:**

Derivation of hESC lines succeeded from poor quality and good quality embryos in the same extent. In several blastocysts, no real inner cell mass (ICM) was seen, but permanent well growing hESC lines could be established. One tripronuclear (3PN) zygote, which developed to blastocyst stage, gave origin to a karyotypically normal hESC line.

**Conclusion:**

Even very poor quality embryos with few cells in the ICM can give origin to hESC lines.

## Introduction

Early embryos at different stages of development have been used for derivation of new hESC lines. The most common isolation of ICM has been from 5-8-day-old blastocysts, but 9-day-old blastocysts have also been used [1], [2], [3]. HESC lines have also been derived from plating the whole blastocyst without isolation of the ICM [4], [5], [6]. Earlier stage embryos have also resulted in hESC lines [7]. Single blastomeres cultured together in groups either in co-culture with hESC or without co-culture have generated hESC lines [8], [9], [10]. One hESC line has been derived from the only blastomere which survived thawing after cryopreservation of a 4-cell stage embryo [11]. Efficiency of the derivation has been poor from arrested embryos [12]. In 2008, Lerou et al. [13], [14] compared derivation of hESC from one-cell arrested embryos to the blastocyst stage, and came to the conclusion that poor quality embryos can generate hESC lines (1/171), but that derivation efficiency significantly correlates with embryo status and that the embryos that have reached the blastocyst stage to a much greater extent generate hESC lines (8/94).

Liu et al. reported a derivation efficiency of 2.4% (4/166) when calculated from day-three embryos. When derivation efficiency was calculated from day-five blastocysts, it was significantly higher (12.5%) [15].

In 2006, Stephenson et al. [16], [17] first proposed that all research groups deriving hESC lines should have a common consensus standard for reporting established hESC lines. The derivation methods and embryo quality and therefore hESC lines can then be more easily compared.

Only poor-quality embryos on days two or three, with a small likelihood of surviving freezing and thawing and generating a pregnancy, have been donated to stem cell research in our hospital. Good-quality embryos frozen for the maximum five-year period which is allowed by the Swedish law have also been donated by couples no longer interested in using them for fertility treatments.

In this analysis, we found that we could derive hESC lines from very poor-quality embryos as frequently as from good quality embryos. A 3PN zygote also gave rise to a karyotypically normal hESC line.

## Materials and Methods

We obtained approval from the Ethics Committee of Karolinska Institutet for derivation, characterisation, and early differentiation of hESC lines from donated supernumerary embryos. Blastocysts were obtained as donations from infertile couples undergoing in vitro fertilisation treatment at our Fertility Unit. Both partners signed an informed consent form after receiving verbal and written information. Only embryos that could not be used in infertility treatment were used in stem cell line derivation. No payment was made to the donors. Good quality embryos have only been donated to stem cell derivation if they have been frozen for the maximum of five years allowed by the Swedish law. Most of the donated fresh embryos with a high score at the time of ICM isolation were slow in development and were therefore not regarded as suitable for IVF treatment. These couples always had good-quality embryos which were transferred as their infertility treatment.

A total of 234 embryos were donated and included in this study between 2002 and 2009. From these embryos we managed to derive 30 permanent fully characterised hESC lines, and 29 early lines which did not continue to grow. The first four derivations were carried out using foetal calf serum (FCS) in the derivation medium [2]. The following 26 hESC lines have been derived and cultured in serum replacement (SR), (Invitrogen, Gothenburg, Sweden) containing media [18]. All the 30 fully characterised lines [19] were included in the EU hESC Registry [20]. An additional 20 ICM derivation attempts were made in a xeno-free medium, which contained X-VIVO 10 (Cambrex Bio Science Walkersville, Inc. Walkersville, MD) as a supplement. Two early hESC lines were obtained, but none of them continued to grow beyond passage two.

### Embryo cultures

Two different embryo culture media were used. After aspiration, the oocytes were collected in ISM1 (Medicult, Jyllinge, Denmark) or G1 (Vitrolife, Gothenburg, Sweden) and incubated for 4–6 hours before insemination by IVF or ICSI. Oocyte and embryo culture was performed in 20 ml drops of culture medium under paraffin oil (Ovoil, Vitrolife) in an atmosphere of 6% CO_2_ in air at 37°C. Pronuclear formation was evaluated 16–18 hours post-insemination (hpi), and only zygotes displaying two pronuclei were selected for embryo transfer. The embryos were cultured from the day of fertilisation up to day three in ISM1 or G1 and from day three to blastocyst stage in ISM2 (Medicult) or G2 (Vitrolife). Evaluation of the embryo quality was performed at 200 times magnification using an inverted microscope equipped with differential interference contrast optics, at 42–44 hpi and at 66–68 hpi. A modified scoring system first described by Mohr et al. [21] was used to score and select embryos for transfer and cryopreservation. A maximal score of 3.5 was given to the embryo if no factor reducing embryo quality was observed. The score was reduced in increments of 0.5 for every reducing factor observed. The reducing factors are; non-ideal numbers of blastomeres (four cells on day two and eight cells on day three were considered ideal), or the presence of more than 10% fragmentation, non-spherical blastomeres, unequal size of blastomeres, uneveness of the cell membrane, cytoplasmic abnormalities or if the embryo did not fill the zona pellucida.

Embryos with multi-nuclear blastomeres were not used for IVF treatment but they could be donated for stem cell derivation.

Embryos scoring 3.0–3.5 were regarded as of top quality, those with a score of 2.5 were considered good quality, and 4–8 cell embryos with a score of at least 2.0 were considered suitable for cryopreservation.

**Figure 1:**
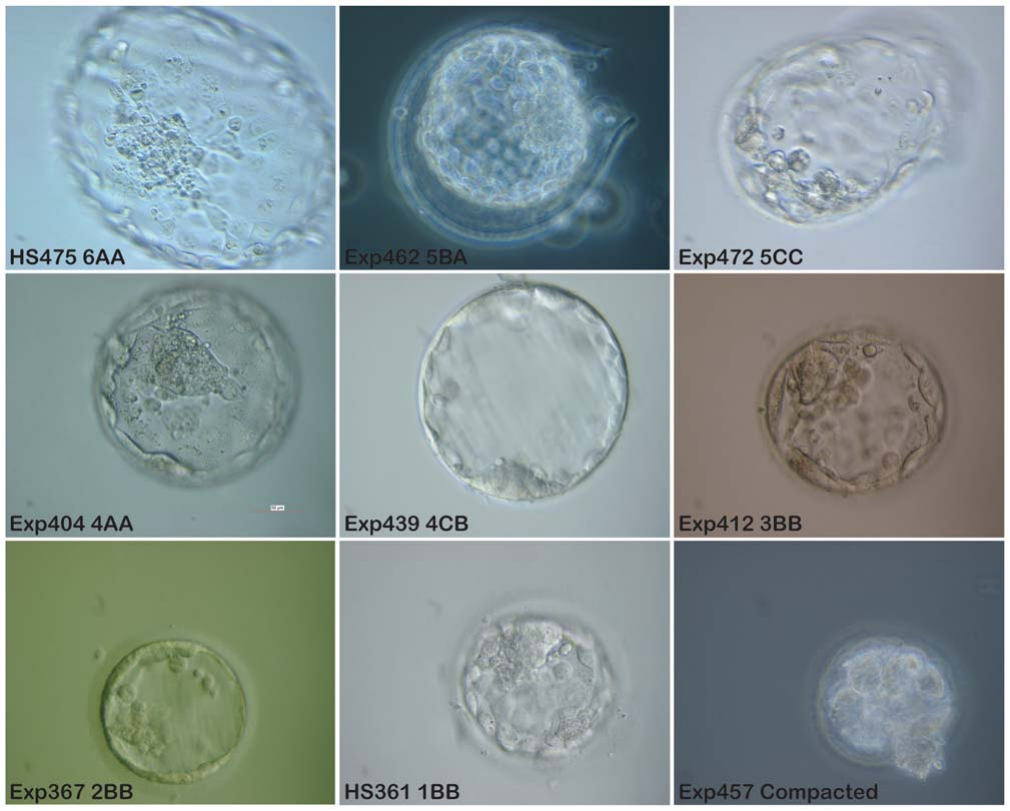
Photographic examples representing the different scores of blastocysts donated for stem cell derivation. **HS475**. Fully hatched blastocyst with clear tightly packed ICM. This embryo generated a new hESC line, HS475. **Exp462**. Hatching blastocyst with average number of cells in ICM, even trophectoderm layer. **Exp472**. Hatching blastocyst. Trophectoderm layer is uneven and the cells in the ICM are few in number and loosely packed. **Exp404**. Fully expanded blastocyst with clear tightly packed ICM and even trophectoderm layer. **Exp439**. Fully expanded blastocyst, with thin zona pellucida. Small ICM and trophectoderm cells of different size. **Exp412**. Fully developed blastocoel. Zona pellucida is still thick, meaning that the blastocyst is not fully expanded. **Exp367**. Early blastocyst. **HS361**. Early blastocyst with blastocoels filling less than 50% of embryo volume. This embryo generated the hESC line HS361. **Exp457**. This embryo is still compacted and has not reached blastocyst stage. All pictures were taken with a 40x objective, phase contrast or DIC, scale bar 50 µm. (Exp =  experiment number. HS is used as a prefix for established hESC lines at Karolinska Institutet).

Selection of blastocysts for transfer or cryopreservation was performed at the IVF unit using the system described by Gardner et al. [22]. The blastocoel was graded from one to six as follows: (1) early blastocyst with a blastocoel of less than 50% of embryo volume; (2) early blastocyst with a blastocoel of 50–80%; (3) fully developed blastocoel of at least 80% of embryo volume; (4) expanded blastocyst; (5) hatching blastocyst; (6) fully hatched blastocyst. The inner cell mass was graded as follows: (A) many cells and tightly packed; (B) average number of cells; (C) few cells and loosely packed. The trophectoderm was graded; (A) for many cells, equal in size and (B) for uneven cells and (C) for few cells. Expanded blastocysts with a good inner cell mass and trophectoderm, i.e. at least 4AB were considered to be of high quality. Blastocysts scoring at least 3AA were considered to be of good quality. For an example of embryos of different scores, see Fig. 1. Photographs of 25 of the donated embryos resulting in new hESC lines are shown in Supporting Fig. S1. More pictures of embryos of different scores not resulting in new hESC lines can be available upon request.

Surplus embryos donated for stem cell research were transferred at the blastocyst stage from the IVF unit at Karolinska University Hospital Huddinge, Sweden, to the stem cell laboratory in the same hospital for hESC derivation.

### Isolation of the ICM

Isolation of the ICM using immunosurgery was carried out for the first 20 hESC derivations [2] using 126 blastocysts. The zona pellucida was first removed by using 0.5% Pronase (Sigma-Aldrich Co., St Louis, USA) and the trophectoderm was removed by immunosurgery as described earlier [23], using rabbit antihuman whole serum (Sigma) and guinea-pig complement serum (Sigma).

**Figure 2:**
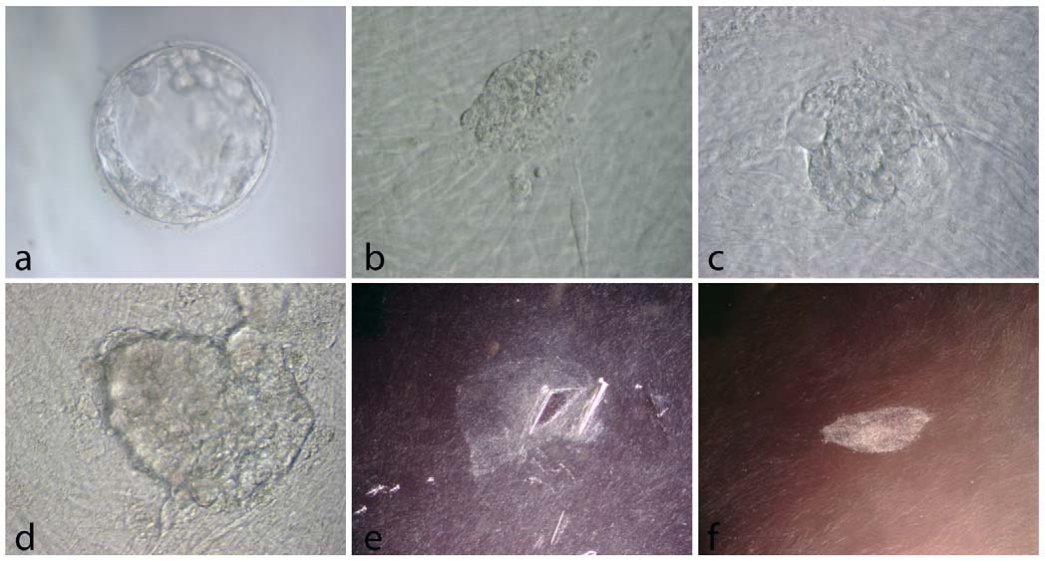
The successful derivation of the hESC line HS429, derived from a 3PN embryo. (**A**) The blastocyst with the score 4CB before inner cell mass isolation. (**B**) The mechanically isolated inner cell mass on human fibroblast feeder layer. (**C**) One day after isolation the inner cell mass has attached. (**D**) Outgrowth four days after isolation. (**E**) Lower magnification of the outgrowth 16 days after inner cell mass isolation, now with the typical stem cell colony morphology. From this outgrowth a small piece had been mechanically passaged four days earlier. (**F**) A colony of the hESC line HS429 at passage two. (Original magnification A–D 40x and E–F 400x). Normal karyotype 46,XX of the line HS429 at passage eight.

For the following 108 blastocyst the ICMs were isolated using a specially made flexible metal needle with a diameter of 0.125 mm, made for us at the Helsinki School of Micromechanics (Espoo, Finland) and now commercially available from Hunter Scientific (Essex, UK). One needle was used to hold the blastocyst while cutting the ICM out with a second needle, as described earlier [3]. The ICM was allowed to grow on human skin feeder cells for 12–15 days before the first transfer to a new feeder plate. For an example of a successful establishment of a new hESC line see Fig. 2.

Human foreskin fibroblasts (CRL-2429; ATCC, Manassas, VA) [2] were used as feeder cells. They were mitotically inactivated by irradiation (40 Gy), and 100,000 cells were plated onto 2.84 cm^2^ dishes (Falcon) overnight to form a confluent monolayer.

The medium for culture of the feeder cells consisted of Iscove's medium supplemented with 10% FCS and 0.5% penicillin-streptomycin (all from Gibco Invitrogen Corporation). We also tested the use of human serum for the feeder cells, and they attached and grew similarly.

### HESC culture medium

The medium used for derivation and culture of the first four established hESC lines consisted of Knockout Dulbecco's modified Eagle's medium (GibcoBRL, Life Technologies), supplemented with 2 nmol/l L-glutamine, 20% FCS (R&D, Sweden), 0.1 mmol/l b-mercaptoethanol (Gibco), 1% non-essential amino acids (Gibco), and recombinant human LIF, 1 ml/ml (Chemicon, UK). Since 2003 all derivations have been carried out in Knockout Dulbecco's modified Eagle's medium supplemented with 20% Knockout SR, 2 mM Glutamax, 0.5% penicillin-streptomycin, 1% non-essential amino acids (all from Gibco Invitrogen Corporation), 0.5 mM 2-mercaptoethanol (Sigma) and 8 ng/ml bFGF (R&H Systems, Oxon, U.K.) [18].

Twenty ICM isolations were carried out in a third medium, supplemented by X-Vivo 10.

### Propagation of the lines

The hESC lines were propagated as described earlier [18], [24]. Splitting of the hESC colonies was performed mechanically at 6-8-day intervals. Colonies were cut into smaller pieces (approximately 6–8) and then transferred onto fresh feeder cells. For passaging, only non-differentiated cells were chosen, as judged morphologically.

### Characterisation of new hESC lines

All new hESC lines were cryo-preserved as soon as sufficiently large cell numbers were attained, at passage level two to ten. The lines were stored in the master bank, and characterised [2], [3], [18], [19]. They have been used in a large number of research projects by many laboratories.

For characterisation, expression of pluripotency markers such as Oct-4, Nanog, SSEA-3, SSEA-4, TRA-1-60, TRA-1-81 and SSEA-1 have been analysed using immune cyto-chemistry and RT-PCR [2], [3], [18], [19], see supporting Fig. S2. Microarray expression analyses by Affymetrix two-cycle GeneChip (Affymetrix, Santa Clara, CA) have been carried out [25], [26], and single nucleotide (SNP) analyses have been run from two of the lines [27], [28].

For the testing of pluripotency, the hESC were differentiated into embryoid bodies by culturing colonies of hESC as spheres in low adhesion culture dishes in SR-containing medium, without the addition of bFGF, for three weeks. They expressed components of all three germ layers, bone morphogenetic protein-4 (BMP-4) for mesoderm, nestin and sex determining region Y-box (SOX-1) for ectoderm and alpha-fetoprotein (AFP) for endoderm.

Pluripotency was also tested *in vivo* by the formation of teratoma in SCID beige mice as described earlier [18]. In brief 10^3^ to 10^4^ hESC were washed twice in D-PBS and put in an 1.5 mL collection tube together with 80 µL culture medium and subsequently implanted beneath the testicular capsule of a young (7-week old) severely combined immunodeficient/beige male mouse (C.B.-17/GbmsTac-scid-bgDF N7, M&B, Ry, Denmark). Teratoma growth was determined by palpation every week and mice were killed (cervical dislocation) eight weeks after implantation. Three to five animals were injected using each hESC line. The teratomas were fixed, and sections were stained with hematoxylin and eosin to analyse the presence of tissue components of all three germ layers, see supporting Fig. S2.

Karyotype analyses were performed by G-banding. Samples of hESCs were treated with colcemid KaryoMAX (0.1 µg/ml;Gibco) for 5 h, trypsinised and treated for 10 min with a hypertonic solution (0.0375 M KCl) and fixed in a 3∶1 methanol and acetic acid solution. Metaphase spreads were G-banded by brief exposure to trypsin and stained with 4∶1 Gurr's/Leishmann's stain (Sigma-Aldrich). A minimum of 10 metaphases were analysed [19].

### Statistical analysis

Logistic regression analysis was performed to assess the association between number of cells on day two and three, morphological score day two and three, ICM score, trophectoderm score and expansion status with the success of establishing a new hESC line. As prior knowledge was sparse regarding the chosen predictors and their relationship to the success of establishing a new hESC line, a backward selection procedure was performed. P-values less than 0.1 were used as the exclusion criterion.

Generalised additive models (GAM) was used to evaluate the functional form of the relationship between each predictor and the probability of successfully establishing a new hESC line. All variables displayed a nonlinear relationship to the outcome and were therefore categorised prior to the logistic regression analysis. Cells on days two and three were classified as (<4 cells,  = 4 cells, >4 cells) and (<8 cells,  = 8 cells, >8 cells) respectively. Morphological score day two and three were both classified as, <2, 2–2.5, >2.5. ICM score, trophectoderm score and expansion status were classified as (0–1, 2, 3), (0–1, 2, 3) and (1–3, 4, >4), respectively.

The statistical analysis was performed in SAS version 9.3 (SAS Institute inc., Cary, NC, USA). Descriptive statistics was done in SPSS 18.0 (SPSS Inc., Chicago, IL).

## Results

### Established hESC lines

We have derived 59 hESC lines since 2002, using 234 early embryos. Thirty of these hESC lines continued to grow and are fully characterised and banked (Table 1). Among these 30 permanent lines we have two lines from two sibling embryos, and three lines from another set of sibling embryos.

**Table 1:** Data from all our 30 established hESC lines at Karolinska Institutet.

hESC line	Derivation day	Derivation method	Score D2	Score D3	Score D5	Score D6	Score D7	Score D8	Expansion status	Score ICM	Score Throphectoderm
**HS 181**	6	Immunosurgery	2C 2,0	4C 2,0	COMP	4BB			4	B	B
**HS 207**	7	Immunosurgery	3C 2,5	6C 1,5	COMP	2CB	2CB		2	C	B
**HS 235**	6	Immunosurgery	7C 2,0	10C 2,5	COMP	3CB	3CB		3	C	B
**HS 237**	6	Immunosurgery	4C 2,0	8C 1,5	1AB	3BB	3BB		3	B	B
**HS293**	6	Immunosurgery	2C 1,0	4C 1,5	1	4BB			4	B	B
**HS306**	5	Immunosurgery	4C 2,5	8C 2,5	3BB				3	B	B
**HS346**	7	Immunosurgery					4BB		4	B	B
**HS351**	6	Immunosurgery	6C 1,5		3AB	4AB			4	A	B
**HS360**	6	Immunosurgery	2C 1,5	4C 3,0	COMP	4CB			4	C	B
**HS361**	7	Immunosurgery	4C 2,0	8C 2,5	MORULA	1	4CB		4	C	B
**HS362**	6	Immunosurgery	2C 2,5	5C 1,5	2BB	4BB			4	B	B
**HS363**	6	Immunosurgery	3C 2,5	5C 2,5	4AC	4AB			4	A	B
**HS364**	6	Immunosurgery	3C 2,0	4C 2,0	COMP	4BB			4	B	B
**HS366**	6	Immunosurgery	2C 2,5	5C 2,0	COMP	3BB			3	B	B
**HS368**	7	Immunosurgery	5C 2,0	2C 1,0	COMP	2CB	4CB		4	C	B
**HS380**	6	Immunosurgery	4C 3,0	7C 2,0	4CB	4BB			4	B	B
**HS382**	6	Immunosurgery	4C 3,5	9C 2,5	2AB	4BB			4	B	B
**HS400**	6	Immunosurgery				4AB			4	A	B
**HS401**	6	Immunosurgery	3C 1,5	6C 2,5	2CB	3AB			3	A	B
**HS402**	6	Immunosurgery	5C 1,5	6C 1,5	2BB	4AA			4	A	A
**HS415**	6	Mechanical Isolation	4C 2,0	8C 3,5	4CB	5			5	C	B
**HS420**	6	Mechanical Isolation	4C 2,0	5C 1,0	4CB	4BB			4	B	B
**HS422**	6	Mechanical Isolation	1C	4C 2,0	COMP	4BC			4	B	C
**HS426**	8	Mechanical Isolation	4C 3,0	8C 3,0	COMP	2BB		5	5	B	B
**HS429**	6	Mechanical Isolation				4CB			4	C	B
**HS475**	7	Mechanical Isolation	5C 2,5	8C 3,5	COMP	2CC	6AA		6	A	A
**HS480**	6	Mechanical Isolation	5C 2,5	8C 2,5	4BC	6BC			6	B	C
**HS481**	6	Mechanical Isolation	5C 1,5	10C 1,5	COMP	3AB			3	A	B
**HS491**	6	Mechanical Isolation	4C 3	5C 3	COMP	4BC			4	B	C
**HS539**	5	Mechanical Isolation			5BB				5	B	B

COMP =  Compacted, C =  cells, D = day, hESC =  human embryonic stem cell, ICM =  inner cell mass.

**Figure 3:**
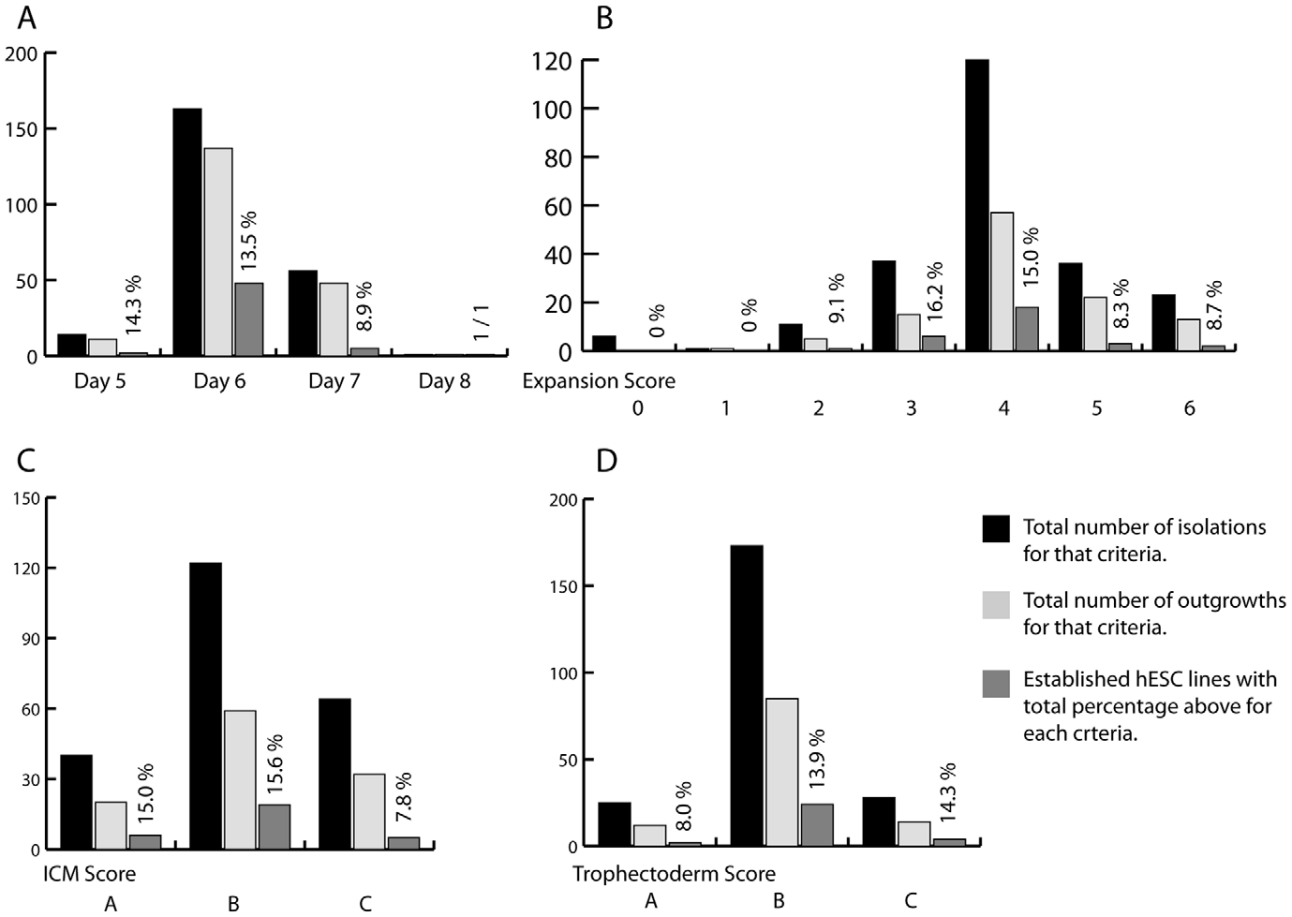
Summary of blastocyst scores and efficiency in derivation. Percentage of successful establishment of permanent hESC lines, for each criteria, is includes above the staple for established hESC lines. (**A**) Day of inner cell mass (ICM) isolation, number of isolations performed on embryos at different days after fertilisation, number of outgrowths from these embryos and the number of successful derivations of hESC lines. (**B**) Expansion Score. Number of isolations performed on embryos with different expansion scores, number of outgrowths and successful derivations. (**C**) ICM Score. Number of isolations performed on embryos with different ICM scores, number of outgrowths and established hESC lines from these embryos. (**D**) Trophectoderm Score. Number of isolations performed on embryos with different expansion scores, number of outgrowths and established hESC lines.

The quality of the embryos, the number of outgrowths and establishment of new lines are shown in Figure 3a–d.

### Morphology and developmental rates of the embryos by days two and three after fertilization

The early embryos resulting in pluripotent hESC lines had between one and seven cells when counted on day two (see Table 1) and the score varied between 1.0 and the maximum of 3.5 on days two and three. On day three the numbers of cells in the early embryos varied between two cells and ten cells for the embryos resulting in hESC lines.

### Day of ICM isolation

Fourteen blastocysts were used for derivation on day five after fertilisation. Eleven of them (78.6%) resulted in outgrowths. We obtained two new hESC lines from the 14 day-five blastocysts (14%). 163 blastocysts were used for derivation on day six after fertilisation, and 137 of these (84%) resulted in outgrowths. Twenty-two of our 30 hESC lines were obtained from day-six blastocysts. In addition, we obtained two early lines that were lost at passages nine and 13. Fifty-six derivations were carried out on seven-day-old blastocysts and 48 of the derivations (85.7%) resulted in outgrowths. Five new hESC lines were established from the 56 day-seven derivations. For summary of these data see Figure 3a. (For an example of a successful establishment of a new hESC line see Fig. 2, for pictures of the embryos resulting in new hESC lines see supporting Fig. S1).

### Expansion status

Only six early embryos were used for stem cell derivation before reaching the blastocyst stage. They had been arrested at the morula stage. One of the arrested embryos was a preimplantation genetic diagnosis (PGD), Huntington's disease. It was the only non-blastocyst embryo that attached to the feeder layer after removal of the zona pellucida. It did not start to proliferate.

One early blastocyst with the expansion status one (early blastocyst with a blastocoel of less than 50% of embryo volume) was used for isolation of the ICM. This resulted in an outgrowth and was passaged twice. No permanent hESC line could be established, See Figure 3b.

Eleven embryos with the expansion status two (early blastocyst with blastocoel filling 50–80% of the volume of the total blastocyst) were used for ICM isolation. 36.4% (four out of 11) resulted in outgrowths and were further passaged, but only one (9.1%) resulted in a permanent hESC line, HS207 (Figure 3b).

Thirty-eight of all blastocysts had expansion status three: fully developed blastocoel filling at least 80% of embryo volume. Of these 38 blastocysts, 16 (42.1%), resulted in an outgrowth, eight were further passaged, and seven (22.3%) of all the embryos with expansion status three gave rise to permanent hESC lines (Figure 3b).

Of all 234 early embryos included in this study, 117 had the expansion status four (fully expanded blastocysts). Fifty-four of the blastocysts resulted in outgrowths (46.2%) and 32 were further passaged (27.4%). Three early lines stopped growing at passage two, one at passage nine, and one early line at passage 15. Fifteen (12.8% of all blastocysts with expansion status four) resulted in stable, fully characterised hESC lines (Figure 3b).

Thirty-eight derivations were carried out using blastocysts with the expansion status five, meaning that they had started to hatch. Twenty-two of these resulted in outgrowths and 13 could be further passaged two or three times (34.2%). Five permanent hESC lines were established (13.2%) out of all the blastocysts with expansion score five (Figure 3b).

Twenty-three blastocysts with expansion status six, meaning fully hatched, were used for ICM isolation. Thirteen (56.5%) resulted in outgrowth. Four were passaged further (17.4%). One promising outgrowth stopped growing at passage four. Two permanent hESC lines were established, 8.7% of all hatched blastocysts (Figure 3b).

### ICM score

Of all derivations performed, 226 had a complete score for the ICM. Out of these 226 blastocysts, 40 had the score A (17.7%), 122 had the score B (54.0%) and 64 blastocysts had the score C (28.3%). Of all blastocysts with the score A for the ICM, 20 (50%) resulted in an outgrowth and 13 (31%) were passaged further. Seven of these resulted in hESC lines. One stopped growing early, at passage four. The remaining six (15%) have been fully characterised as pluripotent hESC lines (Figure 3c).

Fifty-nine (48.4%) of the 122 blastocyst with ICM score B resulted in outgrowths. Thirty-five (28.7%) of these were passaged at least once. Three early lines stopped growing at passages five, nine and 15. Eighteen permanent hESC (14.8%) lines have been established from blastocysts with the score B for the ICM (Figure 3c).

Sixty-four (27.4%) of all blastocyst had the score C for the ICM. Thirty-two of these (50%) resulted in an outgrowth and were also passaged at least once. Of the 64 blastocysts where the ICM consisted of very few cells, six permanent hESC (9.4%) were derived and characterised (Figure 3c).

### Trophectoderm score

Twenty-five (10.7%) of all isolated blastocysts had the sore A for the trophectoderm layer, 173 (73.9%) had the score B and 28 (11.7%) had the score C. Of the early embryos with trophectoderm score A, twelve (48%) resulted in an outgrowth and seven of these were passaged further and two new hESC line was established (8%) (Figure 3d).

Of the 173 blastocyst with trophectoderm score B, 85 (49.1%) resulted in an outgrowth and 46 (26.6%) were passaged, three early lines died out at passage four, five and nine, and in total 24 permanent hESC lines were established from blastocyst with score B for the trophectoderm (13.9%) (Figure 3d).

Twenty-eight of all early embryos had the throphectoderm score C at the blastocyst stage, meaning very few cells of different sizes. Fourteen (50%) resulted in outgrowths, five were passaged and four (14.3%) resulted in new permanent hESC lines (Figure 3d).

### Three pronuclear zygotes

Two 3PN zygotes were donated to stem cell derivation after reaching the blastocyst stage. Both attached to the feeder layer and one started to grow. This 3PN embryo two days after fertilisation had four cells and a score of 3.0, and on day three had eight cells and a score of 3.0. On day five the embryo was still compacted, but it had reached the blastocyst stage at day six. Eight days after fertilisation the blastocyst had started to hatch and mechanical isolation was performed to isolate the ICM. The derivation resulted in the cell line HS429, which is a karyotypically normal well-growing hESC line.

### Statistical analysis

Complete information on the number of cells and morphological score on days two and three, ICM score, trophectoderm score and expansion status was available for 206 embryos. The only variable that showed a significant predictive value for the probability of successfully establishing a new hESC line was having less than four cells on day two, P = 0.018 (Table 2). No other variables included in the model showed any significance, see supporting Table S1.

**Table 2:** Differences of number of cells day two.

Number of cells day 2	Number of cells day 2	Pr |t|	Odds Ratio	Lower Odds Ratio	Upper Odds Ratio
<4 cells	4 cells	0.0181	0.274	0.094	0.800
<4 cells	>4 cells	0.2540	0.468	0.127	1.731
4 cells	>4 cells	0.3369	1.708	0.571	5.111

Least squares means. Having less than four cells on day two showed a significant predictive value for the probability of successfully establishing a new hESC line.

## Discussion

We managed to obtain 30 permanent and 29 early hESC lines from 234 embryos which could not be used for infertility treatment because of poor quality. Often the reason for excluding them from transfer or cryopreservation was developmental delay. That is the reason why we initially received many relatively good-quality blastocysts for stem cell derivation. The policy in our IVF unit has now been changed. The embryos which are not transferred or cryopreserved at the cleavage stage are further cultured to blastocysts and vitrified for possible infertility treatment in the future.

The difficulties with prediction of developmental competence were discussed earlier [29]. However, the term ‘surplus embryos’ based on the morphology on day three was questioned here, since many of the late and also severely fragmented day-three embryos could still develop further to the blastocyst stage. In our data we can see that also embryos with a poor morphology on day five can ‘recover’ to a quite good morphology on day six.

Several of the hESC lines derived from embryos regarded as being of ‘good quality’ on the day of ICM isolation were regarded as ‘poor quality’ on day five. Both hESC lines, HS181 and HS364, when observed by the IVF embryologists five days after fertilisation were still compacted morulae. Therefore they were transferred to the stem cell laboratory where they were left in culture for one more day. The next day (day six), both embryos had developed into fully expanded blastocysts, with the score 4BB. Two more hESC lines, HS293 and HS400, were donated to stem cell derivation on day five with a score of one. These were kept in culture one more day, and when ICM isolation was performed on day six, HS293 had the score 4BB and HS400 had the score 4AB. As a result of these findings the policy at our fertility unit has changed. Embryos are kept for one more day in the IVF laboratory and cryo-preserved for infertility treatment in the future, if they develop to the blastocyst stage with the minimum score of 2BB. Blastocysts with a lower score than 2BB, including all embryos with a score of C for the ICM, can still be donated to stem cell research. There are several slightly different scoring systems, which are all based on the same criteria. We do not believe that using different numbers in scoring would have influenced our results.

Statistical comparison of embryo quality was difficult because of the high degree of variability. We have received 12 early embryos regarded as top quality by the IVF unit with the score 4AA-6AA. Only one hESC line has been successfully derived from them, HS475. The top quality embryos did not appear to result in new hESC lines more often than the others. Five of our hESC lines were derived from embryos with the score C for the ICM, meaning that the ICM contains only a few cells which are hard to visualise. Apparently only a few pluripotent cells, perhaps only one, are needed for the establishment of a stem cell line.

Only five embryos arrested at the cleavage stage were included in this study. None of them resulted in any outgrowth after plating on feeder cells.

We derived hESC lines from embryos regarded as having too poor quality by IVF standards for cryo-preservation, the lowest score being 2CB (cell line HS207). One blastocyst had 3PN when observed one day after fertilisation. For this reason this embryo was regarded as not being suitable for IVF treatment and was donated for stem cell derivation. Six days after fertilisation the blastocyst was fully expanded. Mechanical isolation of the ICM was performed, and the ICM was plated onto feeders. The result was a well growing hESC line, HS429. When analysed at passage eight it had the normal female karyotype 46XX. The line has grown well and remained karyotypically normal at high passages.

We can only speculate why this 3PN embryo resulted in a hESC line with a normal karyotype. One reason could be that when the early zygote was observed, one day after fertilisation, one polar body had still not been extruded, but that this process happened later on. There is also the possibility of normalisation: that the extra sets of chromosomes had been excluded from metaphase and then discarded by the developing embryo. It has been previously shown that 3PN zygotes can be rescued to heteroparental blastocysts by microsurgical correction [30], [31]. Sathananthan et al. [32] have previously described the development of human dispermic embryos. Ten out of 103 3PN zygotes developed to the blastocyst stage. In a case report in 2003, Kattera and Chen described how microsurgical enucleation of a single pronucleus from three 3PN zygotes was performed, and the embryos were transferred to a woman for fertility treatment. The result was a normal healthy baby [33]. No earlier derivation of hESC has been reported from 3PN embryos.

A high proportion of human oocytes are affected by chromosomal abnormalities, but the vast majority of such embryos are unable to develop to blastocyst stage, fail to implant or culminate in a miscarriage [34]. All our 30 hESC lines have been derived from embryos at blastocyst stage. A recent study came with the conclusion that embryo morphology screening might be a better tool than genetic screening for at least a part of the IVF patients [35]. It is interesting to note that large chromosomal abnormalities such as aneuploidy in hESC lines at low passages are not common. Smaller changes seen in hESCs seem to most often appear due to culture adaptations and they are not inherited from the germ cells [28].

In 2006, Zhang et al. [12] described the derivation of two hESC lines from 15 arrested morula stage embryos, but we were not successful in our six such attempts.

We have experience from deriving 30 permanent, and the additional 29 early lines that have been lost either due to differentiation, lack of proliferation or infection (one) before the line could be cryo-stored. On this basis, we can say that both high-quality and poor-quality embryos, and even those containing three pronuclei, can generate pluripotent hESC lines that grow well. Our success rate in derivation of permanent hESC lines was 12.8% (30/234) in 2001-2009. Here we tried to see if we can foresee the likelihood for an embryo of generating a stem cell line by calculating on the scores from day two, three and on the day of derivation, and the number of cells in the early dividing embryo. We compared the different data sets individually or in combination. The only data of significance was the number of cells on day two. Embryos with fewer than four cells had a significant advantage in derivation success compared to embryos with four cells on day two. This correlation with the number of cells, and the likelihood of generating a new cell line was, however, lost on day three. The likelihood of pregnancy is highest when the embryo on day two has exactly four blastomeres, but this parameter does not anticipate stem cell line derivation success.

Our conclusion is that all embryos donated to research that have reached blastocyst stage can give rise to new hESC lines, and attempts should be made even if the blastocyst has a poor score.

## Supporting Information

Figure S1 Pictures of 25 of the early embryos that have resulted in established hESC lines with the score at the time of derivation.
(TIF)

Figure S2 Panel shows teratoma formation from cell line HS401, A. Neural tissue ectoderm 10X, B. Intestinal endoderm 20X, C Cartilage mesoderm 10X. D. Shows expression of pluripotency markers by immunostaining of cell line HS207.
(TIF)

Table S1 Logistic regression analysis was performed to assess the association between number of cells on days two and three, morphological score on days two and three, ICM score, trophectoderm score and expansion status with the success of establishing a new hESC line. Statistical predictive value was only seen for the number of cells on day two after fertilisation.
(DOC)
