# Supplementary figures and images for: No Relationship between Embryo Morphology and Successful Derivation of Human Embryonic Stem Cell Lines
Source: PLoS One. 2010 Dec 31;5(12):e15329. doi: 10.1371/journal.pone.0015329 (PMC3013107; doi:10.1371/journal.pone.0015329)

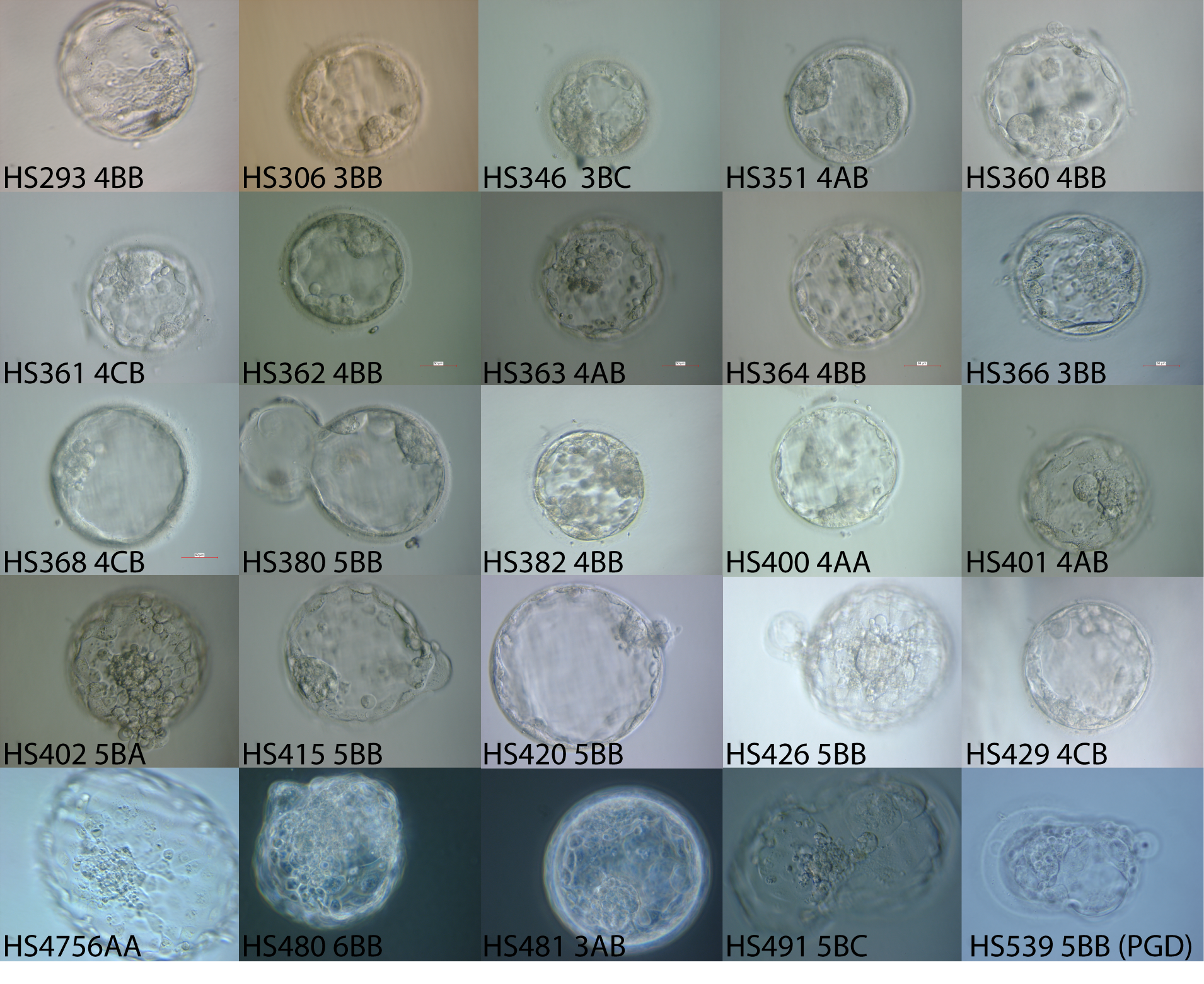

Supplement: Figure S1 — Pictures of 25 of the early embryos that have resulted in established hESC lines with the score at the time of derivation. (TIF) [file pone.0015329.s001.tif]

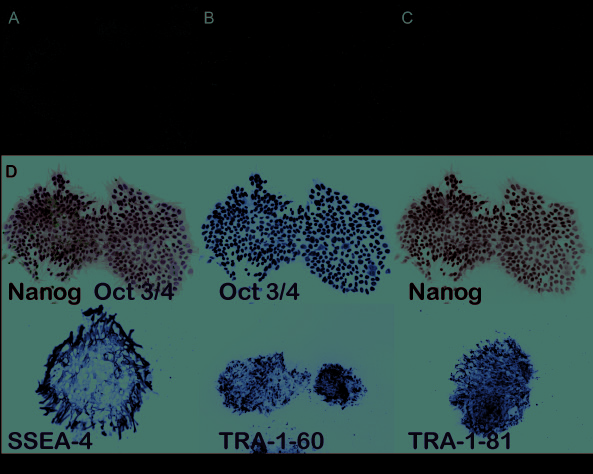

Supplement: Figure S2 — Panel shows teratoma formation from cell line HS401, A. Neural tissue ectoderm 10X, B. Intestinal endoderm 20X, C Cartilage mesoderm 10X. D. Shows expression of pluripotency markers by immunostaining of cell line HS207. (TIF) [file pone.0015329.s002.tif]
