# Supplementary material for: No Relationship between Embryo Morphology and Successful Derivation of Human Embryonic Stem Cell Lines
Source: PLoS One. 2010 Dec 31;5(12):e15329. doi: 10.1371/journal.pone.0015329 (PMC3013107; doi:10.1371/journal.pone.0015329)
Supplement: Table S1 — Logistic regression analysis was performed to assess the association between number of cells on days two and three, morphological score on days two and three, ICM score, trophectoderm score and expansion status with the success of establishing a new hESC line. Statistical predictive value was only seen for the number of cells on day two after fertilisation. (DOC) [file pone.0015329.s003.doc]

| **Number of cells day 2** | **Number of cells day 2** | **Pr ׀t׀** | **Odds Ratio** | **Lower Odds Ratio** | **Upper Odds Ratio** |
| --- | --- | --- | --- | --- | --- |
| < 4 cells | 4 cells | 0.02 | 0.27 | 0.09 | 0.80 |
| < 4 cells | > 4 cells | 0.25 | 0.47 | 0.13 | 1.73 |
| 4 cells | > 4 cells | 0.34 | 1.71 | 0.57 | 5.11 |
| **Number of cells day 3** | **Number of cells day 3** | **Pr ׀t׀** | **Odds Ratio** | **Lower Odds Ratio** | **Upper Odds Ratio** |
| < 8 cells | 8 cells | 0.41 | 1.49 | 0.58 | 3.82 |
| < 8 cells | > 8 cells | 0.47 | 1.62 | 0.44 | 5.97 |
| 8 cells | > 8 cells | 0.91 | 1.09 | 0.26 | 4.57 |
| **Score day 2** | **Score day 2** | **Pr ׀t׀** | **Odds Ratio** | **Lower Odds Ratio** | **Upper Odds Ratio** |
| 0 | 1 | 0.05 | 0.36 | 0.133 | 0.99 |
| 0 | 2 | 0.39 | 0.58 | 0.165 | 2.02 |
| 1 | 2 | 0.40 | 1.60 | 0.539 | 4.75 |
| **Score day 3** | **Score day 3** | **Pr ׀t׀** | **Odds Ratio** | **Lower Odds Ratio** | **Upper Odds Ratio** |
| 0 | 1 | 0.74 | 1.17 | 0.46 | 2.94 |
| 0 | 2 | 0.80 | 0.86 | 0.26 | 2.80 |
| 1 | 2 | 0.59 | 0.74 | 0.24 | 2.27 |
| **Score for ICM** | **Score for ICM** | **Pr ׀t׀** | **Odds Ratio** | **Lower Odds Ratio** | **Upper Odds Ratio** |
| 0 | 1 | 0.68 | 0.81 | 0.30 | 2.20 |
| 0 | 2 | 0.37 | 1.77 | 0.50 | 6.24 |
| 1 | 2 | 0.14 | 2.18 | 0.77 | 6.17 |
| **Score for Trophectoderm** | **Score for Trophectoderm** | **Pr ׀t׀** | **Odds Ratio** | **Lower Odds Ratio** | **Upper Odds Ratio** |
| 0 | 1 | 0.27 | 0.43 | 0.10 | 1.93 |
| 0 | 2 | 0.33 | 0.41 | 0.07 | 2.48 |
| 1 | 2 | 0.95 | 0.97 | 0.31 | 3.05 |
| **Expansion status** | **Expansion status** | **Pr ׀t׀** | **Odds Ratio** | **Lower Odds Ratio** | **Upper Odds Ratio** |
| 0 | 1 | 0.79 | 1.13 | 0.45 | 2.87 |
| 0 | 2 | 0.65 | 1.29 | 0.43 | 3.83 |
| 1 | 2 | 0.80 | 1.13 | 0.43 | 2.97 |
